# 

**Published:** 1888-08

**Authors:** 


### EDITORIAL.

### National Association of Dental Examiners.

The next meeting of this body will be held at Louisville, Ky., on Monday evening, August 27th, at 8 o'clock, and at other times during the week as opportunity may offer and other engagements will permit.

It is very desirble that every State having a law to regulate the practice of Dentistry should be represented: and this for various considerations; first to secure as nearly as possible a uniformity of action and procedure.

SECOND. As a matter of information to the individual Boards, by which they may learn of the efficiency of these laws in the different States; and the best methods of making them most efficient.

THIRD. To do what they may to secure uniformity in the laws of the different States.

FOURTH. To do what they can to aid and stimulate the Dental Colleges so far as practicable to a higher standard of instruction and requirements.

The responsibility resting on this association is very great, and by discrete and well-directed action the good results from its work will be incalculable.

### Annual Meeting of the Dental Faculties' Association.

The National Association of Dental Faculties will meet in a Fifth Annual Session at the Gault House, in the city of Louisville, Ky., at 9 A.M., on Monday, August 27th, 1888.

In order that this meeting may be dispatched before the Southern and American Dental Associations get to work, it is hoped that the entire membership may be represented promptly at the hour indicated. A. O. HUNT, President, Iowa City, Iowa. JUNIUS E. CRAVENS, Secretary, Indianapolis, Ind.

### California State Dental Society.

Will meet in San Francisco on the third Tuesday of August, and continue four days. A very interesting programme has been prepared, which contains the promise of fifteen papers mainly on practical subjects. These are from men of eminence and known ability in the profession, and the bare mention of this fact ought to be sufficient to bring together the entire profession of California; but there will be many other attractions worthy the attention of every member and visitor.

The programme contains the following which it would be well for every State Society, and indeed all other societies as well to follow, viz: "All papers of whatever character, that are to become the property of the association, should be so carefully prepared before their presentation, that they may pass into the hands of the Publication Committee and printer without change."

"That all papers may be uniform, use legal cap, and write in ink on one side only. By so doing you will save much valuable time, annoyance, and labor to the Publication Committee and Secretary. We will hope to have a good report from this meeting."

### American Dental Association.

The American Dental Association will hold its twenty eighth annual meeting at Louisville, Ky. Commencing Tuesday, August 28th, 1888.

GEO. H. CUSHING,
Recording Secretary.

### CONTENTS.

COMMUNICATIONS.

### THE DENTAL REGISTER,

**A MONTHLY MAGAZINE DEVOTED TO THE INTERESTS OF THE**

### DENTAL PROFESSION.

*Terms Reduced to* $2.00 *Per Year. Single Copies,* 25 *Cents.*

### EDITED BY PROF. J. TAFT, D.D.S.

—AND—

### Published by SAM'L A. CROCKER & CO., Ohio Dental and Surgical Depot.

The volume commences in January and closes in December.

The REGISTER being issued monthly is always fresh, therefore Indispensable to the practitioner who desires to keep posted up with the new inventions and improvements which are daily developed. Neither expense nor effort will be spared to give value and variety to its contents. Articles will be illustrated with well executed engravings whenever they will add to the interest or assist to explanation.

Its extensive circulation and frequency of issue make it one of the BEST DENTAL ADVERTISING MEDIUMS in the United States.

All subscriptions must begin with January or July.

Local or special notices, five lines or less, will be inserted for $3 each insertion; over five lines, 50 cts. per line.

All advertising bills payable in advance, or at furthest, after the issue of the first number containing the advertisement. All transient advertisements to be paid for in advance.

☞CONTRIBUTIONS SOLICITED.

Exclusively Editorial Communications should be addressed to the Editor.

The latest number of the REGISTER may be ordered with or without other goods at any time, and all letters should be addressed to

**SAM'L A. CROCKER & CO., Ohio Dental and Surgical Depot, Cincinnati, O.**

